# Comparative Interaction of Pesticides with Microplastics and Soil Organic Matter: A Molecular Simulation Study

**DOI:** 10.3390/toxics14070570

**Published:** 2026-06-29

**Authors:** Fan Zhang, Guoxu Yin, Xibo Lu, Zhuang Wang

**Affiliations:** 1 College of Environmental Science and Engineering, Yangzhou University, Yangzhou 225127, China; mz120251280@stu.yzu.edu.cn; 2 School of Environmental Science and Engineering, Nanjing University of Information Science and Technology, Nanjing 210044, China; 202411120008@nuist.edu.cn

**Keywords:** microplastics, pesticides, soil organic matter, combined pollution, molecular simulation

## Abstract

Microplastics (MPs), a type of emerging persistent pollutant, are attracting increasing attention from both scholars and governments. Agricultural soil is considered an important sink for MPs due to the use of plastic mulches and the application of sewage sludge to fields. Widely used pesticides often coexist with MPs in soils, potentially causing co-contamination that threatens the ecosystem. The interaction between pesticides and soil organic matter (SOM) influences the behavior and toxicity of pesticides in the soil environment, and MPs may participate in this interaction. Preliminary theoretical studies using a combination of molecular mechanics (MM), molecular dynamics (MD), and quantum mechanics (QM) simulations revealed that model MPs and fragments of humic substances (HS) exhibited different adsorption affinities for chlorpyrifos (CPF), a model organophosphorus pesticide, in both vacuum and water. Polypropylene (PP) MPs showed significantly higher adsorption capacity than HS in vacuum, while HS outperformed polyethylene (PE) MPs in water significantly. Furthermore, PP consistently exhibited a significantly higher adsorption capacity than PE regardless of the medium. The adsorption between HS and CPF, as well as between MPs and CPF, was attributed to physical interaction. Furthermore, van der Waals interactions contributed to the mechanism underlying the interactions of MPs/HS with CPF. These findings theoretically demonstrate that MPs can serve as important vectors for the migration of pesticides through the soil system. This work offers a new perspective on the role of MPs in the environmental behavior and toxic effects of pesticides under field-relevant conditions.

## 1. Introduction

Pollution caused by MPs is a great detrimental impact of human activities on the earth’s environment [1]. In the aquatic environment, a large number of MPs are almost everywhere, posing a serious threat to aquatic ecological health [2,3]. While the research done in the terrestrial environment started late, the presence and fate of MPs in soils, including agricultural soils, are attracting increasing attention [4,5,6,7]. MPs can be released into agroecosystems through many human-made processes [8,9,10], such as the application of plastic mulches, irrigation with treated wastewater, and fertilizer from sewage sludge or bio-waste compost. Thus, agroecosystems become a primary entry point for microplastics into terrestrial systems.

Soil is a fundamental resource underpinning ecosystem vitality and human well-being. Inevitably, as a receptor of anthropogenic emissions, it has become a sink for many harmful substances, e.g., pesticides [11], heavy metals [12], and even engineered nanoparticles [13], and is described as their natural shelter and purification place. It is generally recognized that SOM plays an important role in determining the behavior and effects of contaminants [14,15], particularly for soil pesticide pollutants [16,17]. Ordinarily, the presence of SOM decreases the biological activity of coexisting pesticides [18,19]. The role of SOM has both beneficial and adverse aspects. On the one hand, SOM reduces the toxicity of residual pesticides to soil organisms and beneficial plants. On the other hand, SOM also mitigates pesticides’ control effects on the target organisms.

**Figure 1:**
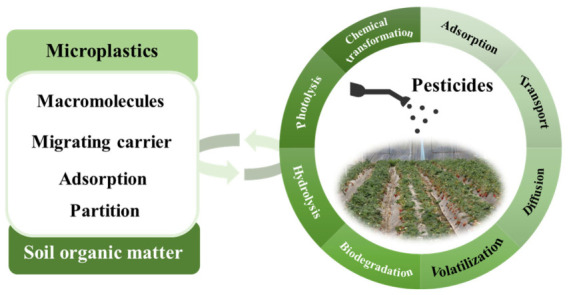
Scheme of the effects of microplastics and soil organic matter on the selective physicochemical behavior of pesticides.

Pesticide pollution is another important environmental concern for humanity. Pesticides usually undergo many physicochemical behaviors (Figure 1), including chemical transformation, photolysis, hydrolysis, biodegradation, volatilization, diffusion, transport, and adsorption. MPs and pesticides can coexist in the soil environment [20]. Because of their small size, large specific surface area, and strong hydrophobicity, MPs in soils are ideal carriers of hydrophobic organic compounds (HOCs) such as pesticides [21,22]. The physicochemical behaviors of pesticides can be altered when MPs are present, indirectly affecting the distribution of pesticides in the soil environment and threatening the health of soil ecosystem. This raises the question of whether concomitant MPs have greater competitive effects than SOM on the behavior of pesticides in the soil environment.

Molecular simulation methods such as MM, MD, and QM simulations have become indispensable tools for studying the environmental behavior of pollutants [23,24]. These calculations bring new insights into typical results that can deepen system knowledge, providing results that cannot be acquired by ordinary laboratories or equipment. CPF is a widely used organophosphorus pesticide [25]. The environmental behavior, fate, and toxicity of CPF have received continuous attention [25,26]. HS are the principal components of SOM. They consist of a complex mixture of naturally occurring organic macromolecules formed through the microbial decomposition and transformation of plant and animal residues [27]. In this work, taking CPF as a model pollutant, we performed a combination of MM and QM simulation studies to investigate the interactions of CPF with HS and two model MPs consisting of PE or PP in both gas and aqueous phases.

## 2. Materials and Methods

The structural model of HS consisting of three fatty acid chains using undecanoic acid (CH_3_(CH_2_)_9_COOH) was cited from part of an initial HS model used by Aquino et al. [28]. The choice of this model was primarily based on computational feasibility, as the inclusion of more realistic and chemically diverse models would significantly increase the computational scale and cost.

Polymer chains derived from five plastic monomers were built as model compounds for MPs, including PE and PP, within the simulation. For the water system, ten water molecules were incorporated in each unit cell. These choices can balance computational accuracy and cost, thereby enabling subsequent calculations at a higher level of theory. All the simulations were carried out in a box with three-dimensional boundary conditions. The periodic model further replicates the polymer chains infinitely in all directions. The dimensions of the simulation box for the interaction between HS and CPF were *a* = *b* = 9.8 Å and *c* = 16.0 Å, with *α* = *β* = *γ* = 90°. The dimensions of the simulation box for the interaction between PE/PP and CPF were *a* = *b* = 8.9 Å and *c* = 16.0 Å, with *α* = *β* = *γ* = 90°. The length of the simulation box in each direction was large enough to enable the interactions between the HS/MP polymer chain and the CPF molecule.

The polymer consistent force field (PCFF) was used to obtain the adsorption behaviors of CPF at the MP/HS interface within the Forcite module [29]. The smart geometry optimization algorithm was used to minimize the energy of the simulation systems. The maximum number of iterations for the Forcite optimization was 1000, which represented an optimal balance (Figures S1 and S2, Supplementary Materials). The cut-off was 12.5 Å. MM geometry optimizations were performed with convergence criteria of 0.001 kcal/mol for energy change and 0.5 kcal/mol/Å for the gradient norm. Three independent MM simulations were performed from three different randomly generated initial complex configurations, and the results are reported as mean values with standard deviations. Statistically significant differences between simulation groups were determined by one-way analysis of variance with the Waller–Duncan post hoc test, at a significance level of *p* < 0.05 (IBM SPSS Statistics for Windows, ver. 23.0, IBM Corp., Armonk, NY, USA).

The optimized systems obtained above were then used for subsequent MD simulations. The MD calculations were performed in the canonical ensemble NVT system in which the number of molecules [N], volume [V], and temperature [T] of the system were kept constant at 298 K. The force field PCFF was used in the simulation framework. The van der Waals interaction cut-off was 12.5 Å, and the Ewald method (accuracy of 0.001 kcal/mol) was used. The simulation was performed for 100 ps, which allowed the studied system to reach equilibrium (Figures S3 and S4, Supplementary Materials), and each step was 1.0 fs. A Nose thermostat was used.

Following the MD simulations, density functional theory (DFT) as a QM simulation method was performed within the Cambridge sequential total energy package (CASTEP) [30]. The CASTEP program was further employed to optimize the isolated individual and complex systems based on the structures obtained from the MM optimization. The functional was set to the generalized gradient approximation (GGA) with the Perdew–Burke–Ernzerhof (PBE) exchange functional [31]. The dispersion-corrected DFT (DFT-D) method with the Grimme vdW correction was adopted to accurately describe weak interactions [32]. The CASTEP optimizations were performed with convergence criteria of 5 × 10^−5^ eV/atom for energy change, 0.1 eV/Å for the maximum force, and 0.005 Å for the maximum displacement. The maximum number of iterations was 2000, which represented an optimal balance (Figures S5 and S6, Supplementary Materials). A detailed analysis of the density of states (DOS) and Mulliken population was conducted following the DFT calculations.

For the interaction systems, interaction energy (*E*_int_) was used to evaluate the stability of the complexes between the MPs/HS and the CPF. The magnitude of *E*_int_ is an indication of the magnitude of the driving force towards complexation. A negative value reflects stable adsorption on the MPs/HS. *E*_int_ was calculated by

*E*_int_ = *E*_MPs/HS-CPF_ − *E*_MPs/HS_ − *E*_CPF_ (1)

where *E*_MPs/HS-CPF_, *E*_MPs/HS_, and *E*_CPF_ represent the energies (total potential energies, van der Waals, and electrostatic) of the complex, the isolated MPs or HS, and the individual CPF, respectively.

## 3. Results and Discussion

**Figure 2:**
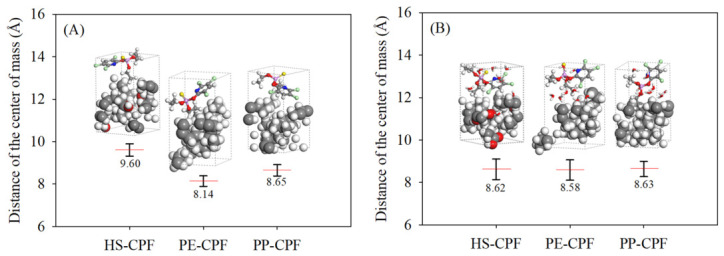
Calculated center-of-mass distances (mean ± standard deviation, *n* = 3) and the stable conformations shown in the insets for chlorpyrifos (CPF) molecules adsorbed on fragments of humic substances (HS), polyethylene (PE), and polypropylene (PP) in vacuum (**A**) and in water (**B**). Gray, white, red, blue, yellow, green, and purple spheres denote carbon, hydrogen, oxygen, nitrogen, sulfur, chlorine, and phosphorus atoms, respectively.

The optimized geometries for the HS-CPF, PE-CPF, and PP-CPF complex systems, in both vacuum (Figure 2A) and water (Figure 2B), were obtained through MM simulations. The center-of-mass distances between CPF and HS/MPs were also computed and are shown in Figure 2. As shown in Figure 2A, the mean distance between MPs and CPF molecules (8.65 Å for PP-CPF and 8.14 Å for PE-CPF) was markedly lower than that observed between HS and CPF (9.60 Å) in vacuum. Overall, this difference was found to be statistically significant (*p* < 0.05). However, in water (Figure 2B), no significant differences were observed in the centroid distances between CPF and the three materials.

**Figure 3:**
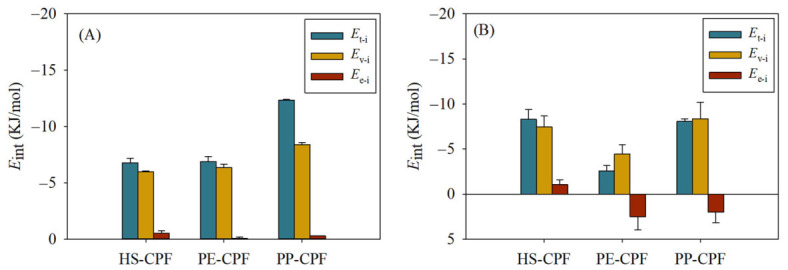
Calculated interaction energies (*E*_int_) derived from total energy (*E*_t-i_), van der Waals interaction energies (*E*_v-i_), and electronic interaction energies (*E*_e-i_) for the chlorpyrifos (CPF) molecules adsorbed on fragments of humic substances (HS), polyethylene (PE), and polypropylene (PP) using the molecular mechanics simulations in vacuum (**A**) and in water (**B**). The data points are mean ± standard deviation (*n* ± 3).

The *E*_int_ values derived from the MM simulation for the HS-CPF, PE-CPF, and PP-CPF complex systems in vacuum and in water are presented in Figure 3. The computed *E*_int_ derived from total energies (*E*_t-i_), van der Waals energies (*E*_v-i_), and electronic energies (*E*_e-i_) between the HS/MPs and CPF are also summarized separately for each energy contribution. The calculated *E*_int_ values in both vacuum and water were negative, indicating that the studied HS/MPs formed stable complexes with the CPF. Moreover, the absolute magnitude of *E*_int_ indicates the strength of the adsorption. As shown in Figure 3A, the *E*_t-i_ and *E*_v-i_ values for CPF with PP were significantly higher than those for CPF with either PE or HS in vacuum (*p* < 0.05). The mean absolute *E*_t-i_ of the complexes decreased in the following order: PP-CPF (12.34 KJ/mol) > PE-CPF (6.90 KJ/mol) > HS-CPF (6.79 KJ/mol). The mean absolute *E*_v-i_ of the complexes followed the same order: PP-CPF (8.41 KJ/mol) > PE-CPF (6.36 KJ/mol) > HS-CPF (5.97 KJ/mol). Therefore, the adsorption strength followed the order PP > PE > HS. Generally, the MPs exhibited relatively high adsorption strength toward the CPF, which was higher than that observed for HS. Furthermore, the *E*_v-i_ contributed 88.01%, 92.17%, and 68.14% of the corresponding *E*_t-i_ for HS-CPF, PE-CPF, and PP-CPF, respectively. By contrast, the contributions of *E*_e-i_ to the *E*_t-i_ values were only 7.81%, 1.09%, and 2.32% for HS-CPF, PE-CPF, and PP-CPF, respectively. Therefore, the *E*_v-i_ constituted a major fraction of the *E*_t-i_ under the vacuum condition.

As shown in Figure 3B, the *E*_t-i_ and *E*_v-i_ values for CPF with PP were also significantly higher than those for CPF with PE in water (*p* < 0.05). However, no significant difference was observed in the interaction energies between the PP–CPF and HS–CPF complexes. The mean absolute *E*_t-i_ of the complexes decreased in the following order: HS-CPF (8.31 KJ/mol) > PP-CPF (8.05 KJ/mol) > PE-CPF (2.55 KJ/mol). The mean absolute *E*_v-i_ of the complexes followed the order: PP-CPF (8.37 KJ/mol) > HS-CPF (7.46 KJ/mol) > PE-CPF (4.44 KJ/mol). In general, HS exhibited an enhanced adsorption capacity for CPF in water, comparable to that of PP and higher than that of PE. Similar to the case in vacuum, *E*_v-i_ made the dominant contribution to *E*_t-i_ in water, compared with *E*_e-i_. From the above results, it can be concluded that the van der Waals interaction considerably contributed to the interaction mechanisms between the HS/MPs and CPF in both vacuum and water.

**Figure 4:**
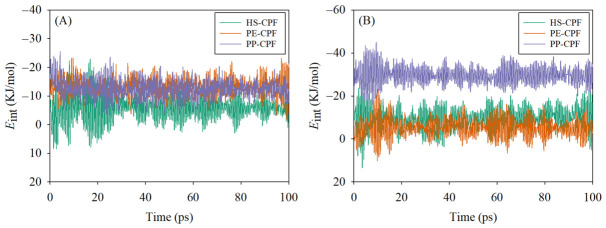
Interaction energies (*E*_int_) derived from total energy (*E*_t-i_) for the chlorpyrifos (CPF) molecules adsorbed on fragments of humic substances (HS), polyethylene (PE), and polypropylene (PP) using the molecular dynamics simulations in vacuum (**A**) and in water (**B**).

The interactions of HS/MPs with the CPF were further investigated by means of MD simulations. The *E*_int_ values derived from the MD simulations for the HS-CPF, PE-CPF, and PP-CPF complex systems in vacuum and in water are presented in Figure 4. As shown in Figure 4A, in agreement with the MM results in vacuum, the time-dependent *E*_int_ of the complexes, as determined by the MD simulations, followed the same decreasing trend: the adsorption strength between MPs and CPF in water was relatively higher than that between HS and CPF. In water (Figure 4B), PP exhibited a stronger and more sustained adsorption capacity for CPF than PE and HS. Similar to the MM simulation results, HS showed enhanced CPF adsorption, which was generally higher than that of PE. Collectively, these results demonstrate that the adsorption affinity of MPs for CPF was notably distinct from that of HS, and such distinction was modulated by both types of MPs and medium conditions.

**Figure 5:**
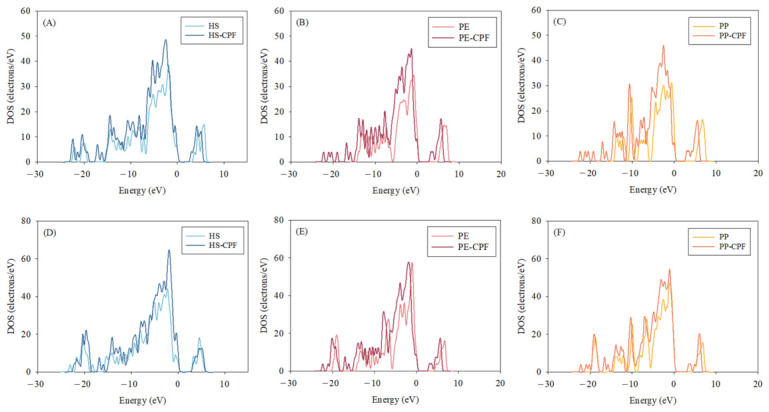
Density of states (DOS) for fragments of humic substances (HS) (**A**,**D**), polyethylene (PE) (**B**,**E**), and polypropylene (PP) (**C**,**F**) before and after chlorpyrifos (CPF) adsorption, based on density functional theory calculations in vacuum (**A**–**C**) and in water (**D**–**F**).

The DOS analysis results of the complex systems of HS and MPs before and after CPF adsorption in vacuum and in water are shown in Figure 5. After CPF adsorption, the DOS of all three materials exhibited significant changes, showing an enhancement compared to that of the pristine materials (without CPF adsorption). Moreover, this trend did not depend on the change in medium conditions. In general, CPF adsorption did not significantly affect the electron density at the Fermi level for any of the three materials, indicating that their electrical properties remained unchanged. Meanwhile, it was noted that a small peak (width < 1 eV) emerged at the Fermi level for both PE (Figure 5B) and PP (Figure 5C) following the CPF adsorption in vacuum, suggesting that dispersion forces could dominate the interactions between the MPs and CPF. In water, the small peak remained for PE (Figure 5E) but showed a tendency to vanish for PP (Figure 5F), indicating that water molecules could exert a negligible influence on the PE–CPF interaction, whereas a more significant effect was observed for the PP–CPF interaction. This further implies that hydrophobic interactions might progressively affect the PP–CPF interaction in aqueous media. The HS model molecules selected in this study contain both hydrophilic and hydrophobic moieties. Combined with the DOS analysis, no new peak was observed at the Fermi level for HS after CPF adsorption in either vacuum (Figure 5A) or water (Figure 5D), suggesting that the HS–CPF interactions in both media are primarily governed by induction and dispersion forces.

Mulliken population analysis was utilized for the elucidation of charge transfer during the MPs/HS-CPF interaction in vacuum and in water, as listed in Table S1. Mulliken population analysis indicated that the *q* values of CPF in its complexes with HS and MPs were less than 0.1 e, suggesting that the adsorption between HS and CPF, as well as between MPs and CPF, was physical in nature. Specifically, CPF carried a negative charge (*q*) in its complex systems with HS in both vacuum and water, indicating charge transfer from HS to CPF during the interaction. After CPF adsorption in water, the amount of *q* transfer from HS was enhanced, suggesting that the strong polarizing field generated by water molecules could promote directional electron transfer to CPF. In the case of PE, the charge state of CPF changed from positive in vacuum to negative in water, indicating that the water environment could increase the electronegativity of CPF. However, CPF remained slightly positively charged when adsorbed on PP in both vacuum and water, revealing that the solvent-induced polarization effect might be inhibited.

In the soil environment, CPF can interact with SOM [33,34]. It was reported that the adsorption capacity of CPF increased with increases in SOM content [35]. For soils with high organic matter contents, the desorption capacity of CPF in soil particles was less than that of adsorption, which reduced the migration ability of CPF in soils. Therefore, when the content of SOM is high, it can adsorb most CPF in soil water, thus effectively reducing the potential ecological threat of CPF application to the soil. Our theoretical calculations revealed that the adsorption affinity of certain MPs (i.e., PP) for CPF exceeded that of HS. These interactions likely arise from physicochemical mechanisms such as van der Waals forces. Given that the fundamental driving force (van der Waals forces) was the same for both HS and MPs, and that MPs exhibited a stronger adsorption capacity, CPF in soil could preferentially desorb from HS and redistribute onto the surface of MP particles. This can make MPs more efficient transport vectors for CPF in the soil environment. Consequently, CPF could migrate over longer distances along with MPs, potentially reaching groundwater or surface water bodies and thereby expanding the scope of contamination. More importantly, the adsorption of CPF onto MPs may alter its bioavailability. On one hand, the sorption of CPF by MPs can reduce its immediate bioavailability, thereby mitigating its short-term toxicity. On the other hand, once MP particles are ingested by soil organisms and enter their digestive tract, CPF may rapidly desorb and be released, leading to a “Trojan horse effect” of MPs, which may cause localized high-dose exposure to CPF and enhance its chronic or acute toxicity. In summary, MPs are reshaping the conventional role of HS as primary adsorbents in soil chemistry, thereby influencing the environmental behavior, fate, and ecotoxicity of CPF.

The present study determined that MPs and CPF exhibited interactions in both vacuum and water, with the strength and nature of these interactions varying among different types of MPs. Previous experimental studies have also confirmed that pristine or aged MPs can adsorb CPF [36,37]. In addition, the differential adsorption of the CPF between PP and PE underscored the role of polymer type in its interaction with MPs. In particular, PP exhibited stronger interactions with CPF than PE. Previous studies have also confirmed that PP particles have a higher adsorption capacity for other pollutants, such as heavy metals [38] and halogenated organic pollutants [39], compared to PE. Mechanistically, van der Waals interactions between PP and CPF were stronger than those between PE and CPF. From a structure–activity relationship perspective, each carbon atom in the PP backbone carries a methyl group (–CH_3_). As an electron-donating group, the methyl moiety slightly increases the electron cloud density on the PP backbone and surface, thereby enhancing surface polarizability. This means the PP surface can more readily generate instantaneous dipoles in response to the electric field of nearby molecules, leading to stronger van der Waals forces, particularly dispersion and induction forces, with the polar groups of CPF. In contrast, the PE backbone consists of repeating –CH_2_– units without side chains. Due to the limited variation in electron cloud distribution, PE exhibits slightly lower overall polarizability than PP. Consequently, the van der Waals interaction strength between PE and CPF was generally weaker than that between PP and CPF. In addition, based on the water contact angle measurements [40,41,42], PP exhibits stronger hydrophobicity than PE. Consequently, the enhanced hydrophobic interaction in aqueous solution could drive the stronger adsorption of CPF onto PP under this study.

However, several knowledge gaps remain. First, there is a need to consider more realistic structures, including well-established humic acid models and longer polymer chains. Second, the study focuses on idealized conditions, whereas real-world scenarios involve competitive sorption from other coexisting substances. Third, aging effects, such as MP surface oxidation or biofilm formation, could alter adsorption behavior over time. Future research will further investigate these factors, including the potential for CPF desorption under varying environmental conditions such as ionic strength, pH, and salinity. Additionally, accurately characterizing the interaction mechanisms will require calculating thermodynamic parameters. Experimental sorption isotherms could also be used to validate computational models and quantify partition coefficients.

## 4. Conclusions

In summary, calculated results of the comparison between model MPs and fragments of HS indicated that the type of MPs and the medium conditions significantly influenced the differential adsorption behavior of MPs and HS toward CPF. From a mechanistic perspective, the adsorption of CPF by both MPs and HS is attributed to physical adsorption, with van der Waals interactions playing a dominant role. Although the simulation results still need more experimental data and should be verified for a wider range of specific types of MPs and SOM, as well as a variety of testing conditions, they provide the first step toward understanding the interaction between MPs and pesticides under field-relevant conditions. Regulatory frameworks for pesticide management should incorporate MP pollution as a contributing factor to environmental persistence and toxicological risks.

## Data Availability

The original contributions presented in this study are included in the article/Supplementary Materials. Further inquiries can be directed to the corresponding authors.

## Supplementary Materials

The following supporting information can be downloaded at: https://www.mdpi.com/article/10.3390/toxics14070570/s1, Figure S1: Variation in the convergence parameters of the HS-CPF (A), PE-CPF (B), and PP-CPF (C) complexes during the Forcite geometry optimization in vacuum. Figure S2: Variation in the convergence parameters of the HS-CPF (A), PE-CPF (B), and PP-CPF (C) complexes during the Forcite geometry optimization in water. Figure S3: Variation of total energy of the HS-CPF (A), PE-CPF (B), and PP-CPF (C) complexes during the MD simulations in vacuum. Figure S4: Variation of total energy of the HS-CPF (A), PE-CPF (B), and PP-CPF (C) complexes during the MD simulations in water. Figure S5: Variation of total energy of the HS-CPF (A), PE-CPF (B), and PP-CPF (C) complexes during the CASTEP geometry optimization in vacuum. Figure S6: Variation of total energy of the HS-CPF (A), PE-CPF (B), and PP-CPF (C) complexes during the CASTEP geometry optimization in water.; Table S1: Mulliken population analysis of the MPs/HS-CPF complexes.
