# Supplementary material for: Comparative Interaction of Pesticides with Microplastics and Soil Organic Matter: A Molecular Simulation Study
Source: Toxics. 2026 Jun 29;14(7):570. doi: 10.3390/toxics14070570 (PMC13417373; doi:10.3390/toxics14070570)
Supplement: Supplementary file 1 [file toxics-14-00570-s001.zip › toxics-4305475-supplementary.pdf]

Supplementary Materials

Comparative Interaction of Pesticides with Microplastics and Soil Organic Matter: A Molecular Simulation Study

Fan Zhang [1]*, Guoxu Yin [1], Xibo Lu [2], and Zhuang Wang [2]*

[1] College of Environmental Science and Engineering, Yangzhou University, Yangzhou 225127, China

[2] School of Environmental Science and Engineering, Nanjing University of Information Science and Technology, Nanjing 210044, China

* Corresponding Authors: Fan Zhang, E-Mail: f.zhang@yzu.edu.cn; Zhuang Wang, E-Mail: zhuang.wang@nuist.edu.cn

Figures 6

Table 1

Pages 8

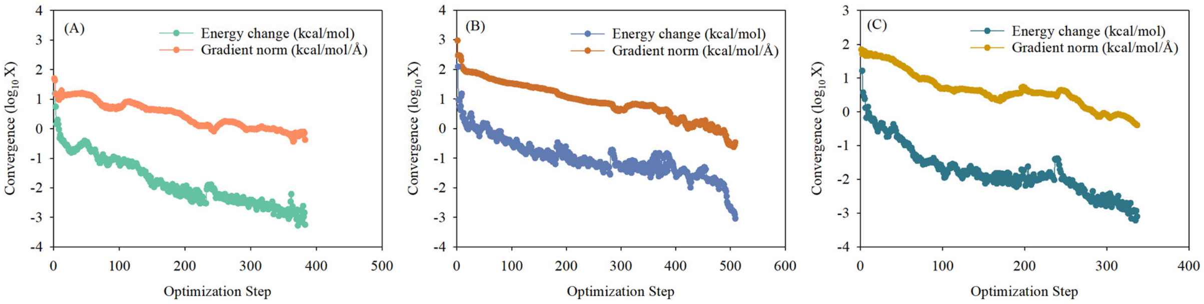

Figure S1. Variation in the convergence parameters of the HS-CPF (A), PE-CPF (B), and PP-CPF (C) complexes during the Forcite geometry optimization in vacuum.

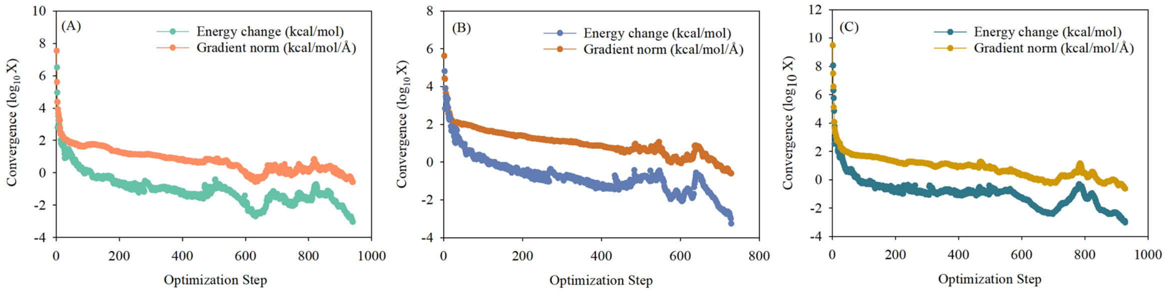

Figure S2. Variation in the convergence parameters of the HS-CPF (A), PE-CPF (B), and PP-CPF (C) complexes during the Forcite geometry optimization in water.

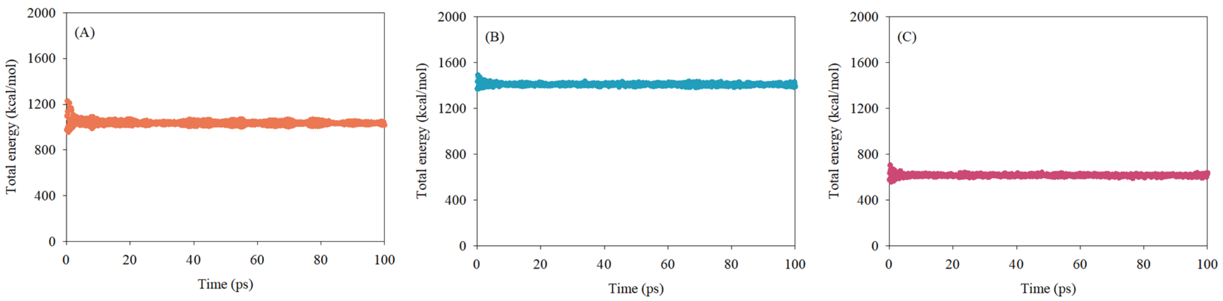

Figure S3. Variation of total energy of the HS-CPF (A), PE-CPF (B), and PP-CPF (C) complexes during the MD simulations in vacuum.

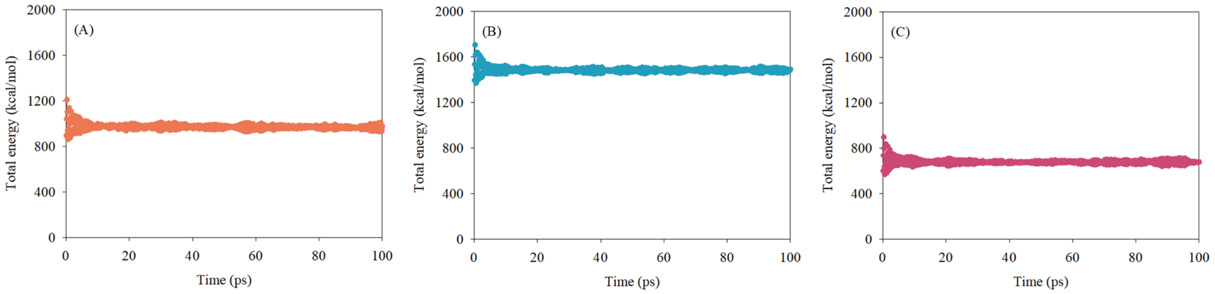

Figure S4. Variation of total energy of the HS-CPF (A), PE-CPF (B), and PP-CPF (C) complexes during the MD simulations in water.

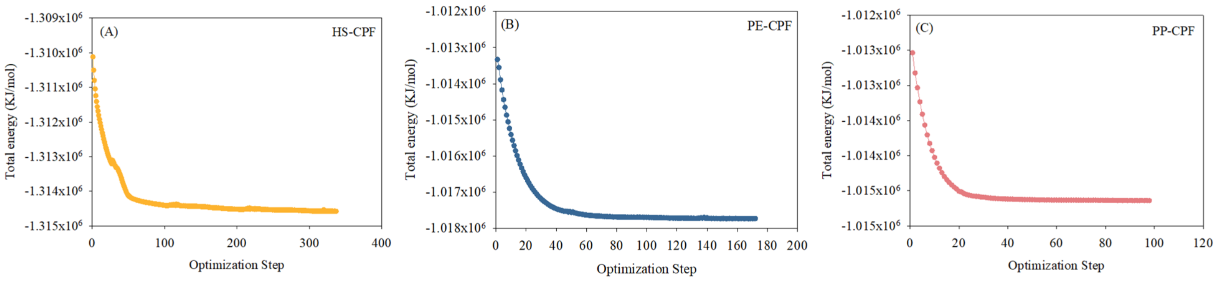

Figure S5. Variation of total energy of the HS-CPF (A), PE-CPF (B), and PP-CPF (C) complexes during the CASTEP geometry optimization in vacuum.

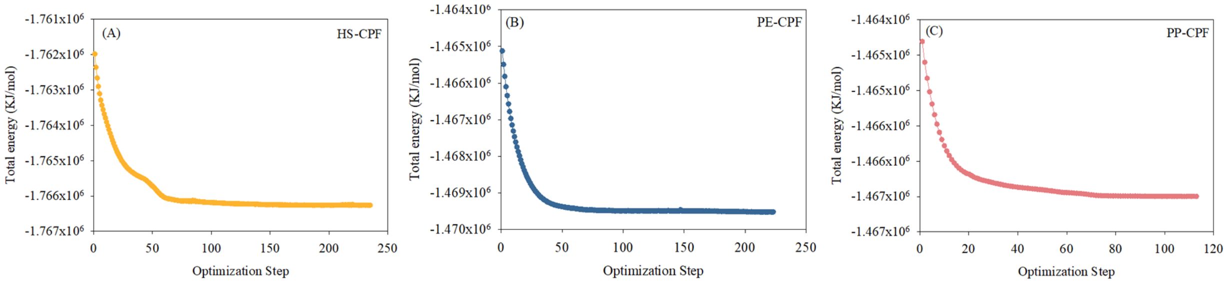

Figure S6. Variation of total energy of the HS-CPF (A), PE-CPF (B), and PP-CPF (C) complexes during the CASTEP geometry optimization in water.

Table S1. Mulliken population analysis of the MPs/HS-CPF complexes

Complexes	CPF electric charge (e)	
	in vacuum	in water
HS-CPF	-0.01	-0.08
PE-CPF	0.04	-0.08
PP-CPF	0.05	0.05
